# Maternal Avoidant and Anxious/Ambivalent Attachment and Children’s Anxiety–Withdrawal: The Serial Indirect Associations of Parental Technoference and Prosocial Behavior

**DOI:** 10.3390/bs16101870

**Published:** 2026-10-10

**Authors:** Ali Özcan, Tuncay Oral

**Affiliations:** Department of Child Development, Pamukkale University, Denizli 20070, Türkiye; aliozcan@pau.edu.tr

**Keywords:** parental technoference, maternal avoidant and anxious/ambivalent attachment, children’s anxiety–withdrawal, prosocial behavior, early childhood

## Abstract

Family-related factors play an important role in children’s socio-emotional adjustment during early childhood. Although maternal insecure attachment has been consistently associated with parenting processes and children’s socio-emotional development, the mechanisms through which these attachment styles are associated with children’s anxiety–withdrawal remain insufficiently understood. Parental technoference and children’s prosocial behavior have been identified as potential mechanisms underlying this association; however, studies examining these variables within an integrated model are limited. This study was conducted to examine the significant serial indirect association of parental technoference and prosocial behavior in the associations between maternal avoidant and anxious/ambivalent attachment and anxiety–withdrawal among preschool children. To test the proposed associations, a mediation model based on structural equation modeling (SEM) was employed. The study sample comprised 608 preschool children attending independent public preschools in the central districts of Denizli, Türkiye, and their parents. Of the participating children, 356 (58.6%) were girls and 252 (41.4%) were boys, ranging in age from 60 to 72 months. The findings indicated that parental technoference and children’s prosocial behavior serially mediated the associations between maternal avoidant and anxious/ambivalent attachment and children’s anxiety–withdrawal. The findings further suggest that reducing parental technoference and fostering children’s prosocial behavior may be relevant to understanding the association between maternal avoidant and anxious/ambivalent attachment and children’s anxiety–withdrawal.

## 1. Introduction

Anxiety-related experiences and withdrawal behaviors are important aspects of children’s socio-emotional adjustment during early childhood. Anxiety generally refers to a state of heightened apprehension or concern about potential negative events, often accompanied by avoidance tendencies and physiological or emotional responses (Creswell & Willets, 2020). During early childhood, elevated anxiety and withdrawal may be reflected in difficulties with emotional adjustment, social engagement, and participation in everyday activities (Rapee et al., 2009). Although anxiety-related behaviors and tendencies toward withdrawal are relevant to socio-emotional development, they may also occur along the broader continuum of socio-emotional adjustment and do not necessarily reflect clinically significant difficulties (Bufferd et al., 2019). Accordingly, examining anxiety–withdrawal in preschool children may provide valuable information about individual differences in socio-emotional adjustment and internalizing tendencies. Understanding factors associated with anxiety–withdrawal during this developmental period is therefore important for identifying family and interpersonal processes related to children’s socio-emotional functioning.

Although anxiety is a natural component of childhood development, its manifestations vary across developmental stages. During the preschool period, anxiety may manifest in forms such as separation anxiety, social anxiety, generalized anxiety, or specific phobias (Egger & Angold, 2006; Şahin & Seven, 2020). The aim of this study is not to assess or explain any specific anxiety disorder; rather, the focus is on anxiety–withdrawal as a dimension of preschool children’s socio-emotional adjustment. Anxiety and withdrawal may be associated with difficulties in children’s emotional and social functioning, including reduced social engagement and participation in everyday activities (American Psychiatric Association, 2013; Connolly & Bernstein, 2007). Anxiety can result from interactions among individual biological predispositions, environmental conditions, and the quality of interpersonal relationships (Frydenberg & Deans, 2016). A lack of consistent experiences that foster feelings of safety and emotional security may be associated with greater vulnerability to anxiety in children (Shaffer & Kipp, 2007), while the relative immaturity of cognitive, linguistic, and emotion-regulation capacities during early childhood may be related to heightened anxiety in the face of uncertainty (Ooi et al., 2017). The literature identifies insecure attachment, parental anxiety, behavioral inhibition, temperament characteristics, traumatic experiences, and parenting practices as factors associated with childhood anxiety. At the same time, social support and effective coping skills have also been associated with lower levels of anxiety (Donovan & Spence, 2000). Furthermore, mother–child interactions, dynamics within the parental relationship, and parental modeling of anxious behaviors constitute important pathways through which anxiety may develop in children (Jones et al., 2015b; Rapee et al., 2019). Thus, anxiety–withdrawal during early childhood may be understood, in part, through family attachment processes, as parents’ internal attachment orientations and the quality of parent–child relationships are important relational factors (Brumariu & Kerns, 2010; Kohlhoff et al., 2023).

Anxiety-related difficulties and withdrawal behaviors emerging during the preschool years may interfere with children’s everyday functioning. They may be associated with less adaptive socio-emotional adjustment across subsequent developmental periods (Steinsbekk et al., 2022). Childhood anxiety may also be associated with difficulties with peer relationships, academic adjustment, school performance, and participation in leisure activities (Essau et al., 2000; Ezpeleta et al., 2001). Persistent anxiety may be associated with the escalation and persistence of behavioral problems and has been linked to underdeveloped social skills and an increased risk of peer rejection (Loeber & Farrington, 2000). Similarly, children who exhibit behavioral problems during early childhood tend to experience greater difficulties across diverse social contexts than their more developmentally well-adjusted peers (Campbell, 1995). These concerns highlight the importance of the preschool and early elementary school years for understanding behavioral and emotional difficulties. Despite growing evidence concerning anxiety in older developmental periods, the early emergence of anxiety and its developmental dynamics during the preschool years remain insufficiently understood (Egger & Angold, 2006). This gap matters given the potential benefits of early identification and prevention efforts. Understanding anxiety processes during the preschool period may therefore provide a basis for identifying factors related to developmental risks (Şahin & Seven, 2020). Accordingly, the present study examines how maternal insecure (avoidant and anxious/ambivalent) attachment is associated with preschool children’s anxiety–withdrawal, with particular attention to the serial mediating roles of parental technoference and children’s prosocial behavior.

### 1.1. Associations Between Maternal Avoidant and Anxious/Ambivalent Attachment and Anxiety–Withdrawal Among Preschool-Aged Children

Adult attachment is conceptualized within the framework of internal working models that develop over time based on individuals’ relational experiences with their primary caregivers during early childhood. These models encompass mental representations of the self and significant others and guide interpersonal functioning across the lifespan (Fraley et al., 2011). Research in adult attachment has increasingly highlighted the relevance of attachment security to individuals’ coping with life stressors and adaptation to changing circumstances. In this context, attachment security is conceptualized as expectations that significant others will be available, responsive, and supportive when needed within close relationships (Mikulincer & Shaver, 2016). Its potential relevance may be particularly evident under prolonged or chronic stress, where it has been associated with more effective coping and psychological adaptation (Berant et al., 2003). Parenting, as a lifelong role involving multifaceted responsibilities and complex relational processes, has become increasingly demanding amid rapid social transformations. The transition to parenthood entails substantial changes in family roles and daily routines, and mothers often disproportionately assume caregiving responsibilities, making the postpartum period a particularly significant life transition (Cowan et al., 1996). Within this framework, maternal attachment patterns may be associated not only with mothers’ psychological well-being but also with their children’s socio-emotional development and psychological adjustment. Empirical evidence suggests that parents’ adult attachment characteristics may be associated with children’s emotional development and behavioral functioning (Adam et al., 2004; Cowan et al., 2009).

Within adult attachment research, individual differences are frequently organized around secure attachment and two insecure orientations. Individuals with secure attachment generally maintain favorable internal models of the self and others, viewing themselves as deserving of affection and relationships as safe, dependable, and emotionally responsive. They can also provide and receive emotional support and express positive and negative emotions flexibly within close relationships (Levy et al., 2018; Shaver & Mikulincer, 2007). In contrast, avoidant attachment is characterized by a negative view of relational dependency and strategies to maintain emotional distance and minimize closeness in significant relationships (Fraley et al., 2011). Anxious/ambivalent attachment is characterized by excessive needs for approval and closeness, heightened fears of abandonment, and intense emotional reactivity in close relationships (Ainsworth, 1978). This orientation is associated with internal working models shaped by uncertainty regarding caregivers’ availability and affection during childhood. Individuals with anxious/ambivalent attachment may therefore exhibit heightened emotional responses, excessive proximity-seeking, and persistent fears of rejection or abandonment. The heightened activation of the attachment system underlying this pattern may foster dependence on external reassurance and interfere with the development of balanced and autonomous relationships (Shaver & Mikulincer, 2002). Consistent with this pattern, anxious/ambivalent attachment has been associated with difficulties in emotion regulation, lower self-esteem, maladaptive coping strategies, and greater vulnerability to anxiety and depressive symptoms (Mikulincer & Shaver, 2016).

From an attachment theory perspective, parents’ early relational models and attachment orientations provide important foundations for their caregiving behaviors and interactions with their children (Bowlby, 1982; Feeney & Woodhouse, 2016). Parents’ attachment-related orientations may be associated with how they engage with their children, organize parenting behaviors, and respond to child-related demands, as well as with maltreatment risk (Berlin et al., 2011; Jones et al., 2015a). Their attachment-based internal representations may also show up in child-rearing attitudes, which shape children’s social functioning and emotional regulation (Eisenberg et al., 2005; Nezhad et al., 2014). Parenting attitudes, in turn, are considered important determinants of children’s well-being and socio-emotional development (Morawska & Sanders, 2011). Emerging evidence therefore suggests that associations between parents’ attachment experiences and children’s behavioral adjustment and adaptation may reflect intergenerational processes. Parents’ insecure attachment orientations have been identified as relational risk factors for children’s adjustment, with anxious and avoidant patterns associated with elevated internalizing and externalizing problems (Cowan et al., 1996; Kohlhoff et al., 2023). Similarly, parents who report more secure relationships with their own caregivers tend to have children who exhibit fewer externalizing behavioral difficulties (Roskam et al., 2011). Externalizing problems include behaviors such as hyperactivity, aggression, and antisocial tendencies, whereas internalizing problems encompass anxiety and depression that may undermine socio-emotional adjustment (Kovacs & Devlin, 1998; Merrell, 2001). Within an attachment framework, mothers’ internal attachment representations may be expressed through interaction patterns with their children, shaping the emotional climate of the parent–child relationship and influencing children’s socio-emotional and psychological development. Among the potential outcomes of these relational processes, anxiety–withdrawal has received particular attention as an indicator of internalizing difficulties. Maternal insecurity may shape caregiving interactions in ways that reduce emotional support and relational stability, potentially increasing children’s susceptibility to anxiety–withdrawal over time. Supporting this proposition, Babaroğlu (2020) reported that parental attachment style, particularly maternal attachment style, was significantly associated with anxiety levels among preschool-aged children.

Although the intergenerational transmission of attachment styles remains debated, Bowlby (1980) proposed that early attachment experiences may be transmitted across generations through individuals’ internal working models with relatively little modification. Evidence suggests an intergenerational pathway of attachment development whereby mothers’ internal attachment representations are reflected in the security of attachment established by their infants (Lippe et al., 2010). Parents may therefore preserve and project internal working models developed through relationships with their own caregivers onto their children, contributing to continuity across generations. From an attachment perspective, internal working models are relatively stable cognitive-affective structures that develop from early relational experiences and are thought to be associated with how individuals perceive, evaluate, and engage in close relationships throughout life; accordingly, they have been considered a core component of personality organization (C. George & Solomon, 1999). Within this context, parental attachment patterns, particularly maternal attachment, have been identified as important determinants of the quality of parent–child attachment relationships (van IJzendoorn, 1995). Viewed through a developmental attachment lens, early parent–child relational experiences may therefore represent an important context associated with children’s anxiety–withdrawal and socio-emotional adjustment. A substantial body of research has identified a moderate and significant relationship between children’s insecure attachment experiences and anxiety–withdrawal (Colonnesi et al., 2011). Similarly, Moss et al. (2006) demonstrated that children who exhibited disorganized attachment patterns between the ages of five and seven showed significantly elevated anxiety–withdrawal over the subsequent two-year period. Collectively, these findings suggest that insecurity in early attachment relationships may place children at greater risk for internalizing problems over time. Children’s observation and internalization of attachment-related parental behaviors further suggest that attachment-related influences may operate through enduring processes of social learning and conditioning. Consequently, parents’ adult attachment patterns and child-rearing attitudes may represent important family and relational factors associated with children’s psychological well-being and interpersonal functioning. Examining the association between maternal avoidant and anxious/ambivalent attachment and anxiety–withdrawal among preschool-aged children is therefore important for understanding the relational processes associated with children’s socio-emotional adjustment.

### 1.2. Associations Between Parental Technoference and Anxiety–Withdrawal Among Preschool-Aged Children

As mobile technologies have become deeply integrated into everyday life, attention has increased to their potential to interrupt parent–child interactions by drawing parents’ attention away from their children and weakening the quality of family relationships (Idoiaga-Mondragon et al., 2025). Consistent with these concerns, sociological and psychological research indicates that mobile technologies may weaken face-to-face social interactions and reduce the quality of interpersonal engagement (McDaniel & Radesky, 2018; J. Radesky et al., 2015). Frequent parental mobile phone use may interfere with responsive caregiving and lead parents to respond more negatively to their children’s behaviors (J. S. Radesky et al., 2014). Technology-related interruptions may also weaken parent–child exchanges by limiting verbal and nonverbal communication and reducing opportunities for emotional connection (J. Radesky et al., 2015). Because secure attachment relationships provide an important foundation for children’s emotion regulation and social competence (Ainsworth, 1978), persistent disruptions in parent–child interactions may place healthy development at risk. Consistent with this perspective, excessive parental smartphone use has been associated with higher levels of anxiety–withdrawal and depressive symptoms in children, potentially undermining their subjective well-being (Xie & Xie, 2020; Di Maggio & Zappulla, 2014).

Children’s social competence is closely linked to parental communication and family interaction patterns. However, increasing digital technology use may disrupt parent–child interactions and parenting practices, leaving individuals physically present but psychologically disengaged from ongoing social exchanges (Knitter & Zemp, 2020). This phenomenon is commonly conceptualized as technoference, referring to interruptions in interpersonal interactions caused by digital and mobile devices. In the parenting context, parental technoference specifically refers to disruptions in parent–child interactions resulting from parents’ excessive engagement with digital technologies (McDaniel & Coyne, 2016; J. S. Radesky et al., 2014). Research indicates that parental technoference is associated with changes in parenting practices and declines in the quality of parent–child interactions (McDaniel & Radesky, 2018; McDaniel et al., 2018). According to Parental Acceptance–Rejection Theory, when parents devote excessive attention to digital devices, children may interpret reduced parental engagement as rejection, neglect, or emotional unavailability, which may negatively affect their self-worth (Rohner & Lansford, 2017; X. Wang et al., 2022). These perceptions may subsequently increase children’s vulnerability to internalizing problems, including anxiety–withdrawal and depressive symptoms (Sundqvist et al., 2020).

The Compensatory Internet Use Model (CIU) proposes that individuals may rely on digital technologies to cope with unmet emotional needs and that negative offline experiences, such as social isolation, loneliness, and stress, may increase engagement in online environments (Kardefelt-Winther, 2014). Consistent with this framework, loneliness, social anxiety, and difficulties in emotion regulation have been shown to explain associations between insecure or anxious attachment patterns and problematic technology use (Young, 1998; Huang et al., 2025; Z. Wang & Xuan, 2024). This perspective is particularly relevant to the present study because it suggests that insecure attachment orientations may be associated with greater reliance on digital technologies, which may in turn interfere with parent–child interactions. Excessive engagement with digital media among parents with insecure attachment patterns may be related to difficulties in parent–child relationships and children’s developmental adjustment. Technology-related attentional distraction can reduce the quality and quantity of verbal and nonverbal communication between parents and children, thereby undermining emotional connectedness (J. Radesky et al., 2015). Such disruptions have been associated with poorer psychological functioning, including higher levels of anxiety–withdrawal, depression, and negative self-perceptions (Ergün et al., 2020). In contrast, healthy parent–child interactions have been associated with lower levels of psychological and behavioral problems (Bireda & Pillay, 2018). Consistent with this view, Abu-Taieh et al. (2022) found that greater parental engagement with digital technologies was associated with increased anxiety –withdrawal and depressive symptoms among parents, which were subsequently linked to higher levels of anxiety and depressive symptoms in their children. Taken together, these findings suggest that parental technoference may represent an important pathway linking parental characteristics and technology-related behaviors with children’s psychological adjustment. In particular, the literature provides a basis for examining whether maternal insecure attachment patterns are associated with greater parental technoference and whether higher levels of technoference are, in turn, associated with greater anxiety–withdrawal among preschool children.

### 1.3. Associations Between Prosocial Behavior and Anxiety–Withdrawal Among Preschool-Aged Children

Prosocial behavior, also conceptualized as sociability, represents a multidimensional construct encompassing a broad range of interpersonal behaviors and social interactions (Walsh et al., 2021). Generally defined as positive social behavior, it plays an important role in initiating and maintaining healthy social relationships. Emerging during early childhood, prosocial behaviors include empathy, sympathy, helping, sharing, cooperation, comforting, and concern for others. Because prosocial tendencies are associated with socio-cognitive and socio-emotional competencies, they may also be associated with greater social integration, adaptation, and interpersonal cooperation (Eisenberg et al., 2015). Research has demonstrated that children’s prosocial behaviors are often guided by intrinsic motivation arising from concern for others’ well-being (Hepach et al., 2013). Developmental studies further indicate that prosocial behavior increases throughout the first two decades of life, with the most pronounced growth occurring during the preschool years (Eisenberg & Fabes, 1998). A growing body of evidence links prosocial behavior to psychological well-being, behavioral adjustment, positive peer relationships, and academic success (Caprara et al., 2000; Swit & McMaugh, 2012). More recent meta-analytic evidence suggests that engagement in prosocial activities is associated with enhanced well-being and reduced negative emotional states (Zhang et al., 2023). Similarly, C. Schwartz et al. (2003) found a significant positive relationship between prosocial behavior and mental health, with helping others showing a stronger association with positive mental health outcomes than receiving help. These findings suggest that active engagement in prosocial behavior may be particularly relevant to psychological adjustment.

Prosocial behaviors are highly valued in society, and the social rewards associated with them may relate to individuals’ emotional well-being (Hay, 1994). Prosocial behavior has also been identified as an important factor associated with depression, anxiety–withdrawal, and other forms of emotional distress (Fujiwara, 2007; Haroz et al., 2013). Engaging in prosocial behaviors may promote stronger social relationships as well as greater psychological well-being and quality of life among those who perform them (C. E. Schwartz & Sendor, 1999). From a positive psychology perspective, prosocial behavior may promote mental health by fostering psychological resources such as optimism, happiness, and self-determination (Csikszentmihalyi & Seligman, 2000; Myers, 2000; Peterson, 2000; Ryan & Deci, 2000). In particular, optimism has been highlighted as a resource that may enhance resilience against anxiety–withdrawal and psychological distress (Peterson, 2000). Accordingly, prosocial behavior may represent an important developmental resource associated with children’s anxiety–withdrawal. Carlo (2014) noted that children who consistently exhibit prosocial behaviors tend to possess well-developed socio-emotional and socio-cognitive capacities, which may strengthen resilience and reduce vulnerability to internalizing and externalizing problems. Prosocial behaviors during early childhood may be associated with more effective coping with developmental risk factors and more adaptive adjustment. Supporting this perspective, prosocial behavior during the preschool period has been associated with lower levels of subsequent internalizing and externalizing problems (Gülseven et al., 2022). Similarly, Zhang et al. (2023) found that prosocial behavior was significantly associated with reduced symptoms of depression and anxiety–withdrawal. Taken together, these findings suggest that children’s prosocial behavior may represent an important factor associated with anxiety–withdrawal during early childhood. The existing literature therefore provides a basis for examining whether greater prosocial behavior is associated with lower levels of anxiety–withdrawal among preschool children and whether maternal insecure attachment patterns may be indirectly associated with children’s anxiety–withdrawal through the sequential roles of parental technoference and children’s prosocial behavior.

## 2. The Present Study

Existing research has provided valuable insights into parental attachment, technology-related disruptions in parent–child interactions, prosocial behavior, and anxiety–withdrawal. However, relatively few studies have integrated these variables into a comprehensive conceptual framework that explains their interrelationships. Accordingly, the present study addresses this gap by testing a structural equation model that examines the associations among maternal avoidant and anxious/ambivalent attachment, technoference in parent–child interactions, prosocial behavior, and anxiety–withdrawal in children. More specifically, we hypothesized that maternal avoidant and anxious/ambivalent attachment would be associated with higher levels of technoference in parent–child interactions and that technoference would be negatively associated with children’s prosocial behavior. We further expected lower levels of prosocial behavior to be associated with higher levels of anxiety–withdrawal among preschool children. Evidence from previous studies indicates that individuals characterized by insecure attachment patterns are more likely to engage extensively with digital technologies, including social media and the Internet, and consequently face a greater risk of problematic technology use and technology addiction (Erözkan, 2011; Monacis et al., 2017). Such excessive engagement with technology may divert parents’ attention away from interactions with their children, diminishing both the frequency and quality of verbal and nonverbal communication—a process conceptualized as technoference (J. Radesky et al., 2015). Research has further shown that technoference in parent–child interactions is associated with lower levels of prosocial behavior among preschool children (Hong et al., 2019; Sundqvist et al., 2020). In addition, prosocial behavior has been linked to maternal attachment styles (Attili et al., 2011) as well as children’s anxiety–withdrawal (Okuyucu et al., 2024), suggesting that it may represent a key mediating mechanism within the proposed model. Building on these theoretical and empirical findings, the present study proposes that maternal avoidant and anxious/ambivalent attachment may be associated with higher levels of technoference during interactions with preschool-aged children, which may, in turn, be associated with lower levels of prosocial behavior in children. Accordingly, it is hypothesized that parental technoference and children’s prosocial behavior may constitute a serial mediating pathway in the association between maternal avoidant and anxious/ambivalent attachment and children’s anxiety–withdrawal levels. In other words, the association between maternal avoidant and anxious/ambivalent attachment and children’s anxiety–withdrawal may be explained through a sequential mechanism in which higher levels of technoference in parent–child interactions are associated with lower levels of prosocial behavior among children, which may subsequently be associated with elevated anxiety–withdrawal symptoms. Within this framework, higher levels of technoference are expected to be associated with lower levels of prosocial behavior, and lower levels of prosocial behavior are expected to be associated with elevated anxiety–withdrawal symptoms in children. Accordingly, the following research questions were developed to examine the proposed conceptual model:

RQ1. Is maternal avoidant and anxious/ambivalent attachment positively associated with anxiety–withdrawal among preschool-aged children?

RQ2. Does parental technoference mediate the relationship between maternal avoidant and anxious/ambivalent attachment and children’s anxiety–withdrawal?

RQ3. Does prosocial behavior mediate the relationship between maternal avoidant and anxious/ambivalent attachment and children’s anxiety–withdrawal?

RQ4. Do parental technoference and prosocial behavior serially mediate the relationship between maternal avoidant and anxious/ambivalent attachment and children’s anxiety–withdrawal?

## 3. Materials and Methods

### 3.1. Participants

The present study adopted a cross-sectional design based on a quantitative research approach. Participants were selected through convenience sampling, a non-probability sampling technique that allows voluntary participation and supports efficient data collection within a limited timeframe (Fraenkel et al., 2012). The study included 608 preschool children attending independent public preschools in the central districts of Denizli, Türkiye, and their mothers. Among the participating children, 356 (58.6%) were girls, and 252 (41.4%) were boys. In terms of age, 253 children (41.6%) were five years old, whereas 355 (58.4%) were six years old. Regarding maternal education, 97 mothers (16.0%) had completed primary school, 94 (15.5%) had graduated from middle school, 174 (28.6%) had completed high school, 218 (35.9%) held a bachelor’s degree, and 25 (4.1%) had earned a postgraduate degree. Fathers’ educational attainment showed a similar distribution: 72 (11.8%) had completed primary school, 98 (16.1%) had graduated from middle school, 212 (34.9%) had completed high school, 185 (30.4%) held a bachelor’s degree, and 41 (6.7%) possessed a postgraduate degree or higher. With respect to socioeconomic status, 129 families (21.2%) identified themselves as belonging to the low socioeconomic group, 448 (73.7%) to the middle socioeconomic group, and 31 (5.1%) to the high socioeconomic group.

### 3.2. Procedure

Before data collection commenced, ethical approval was granted by the Social and Human Sciences Research Ethics Committee of Pamukkale University (Approval No. 68282350/2026/01; 22 January 2026). All study procedures complied with the ethical principles of the 1975 Declaration of Helsinki and established guidelines for scientific research involving human participants. Prior to administering the study instruments, preschool teachers were informed about the objectives and procedures of the research. They were also notified that participation was entirely voluntary and received detailed instructions regarding the administration of the assessment measures. Mothers completed the Adult Attachment Style Scale and the Parent–Child Technoference Scale, whereas teachers completed the remaining questionnaires. Written informed consent was obtained from all participants before their inclusion in the study. Data were collected using an online survey platform, which allowed participants to discontinue their participation at any point without consequence. To reduce the likelihood of missing responses, the survey system required all questionnaire items to be completed before submission was permitted. A total of 643 cases were initially obtained. During the data-screening process, 22 cases were excluded because the participants failed the attention-check item included in the survey. The attention-check item was a simple question that could be readily answered by all participants, such as “What is the capital of Türkiye?” An attention-check item was included in both the scales completed by teachers and those completed by mothers. The remaining 621 cases were then screened for multivariate outliers using Mahalanobis distance based on the five variables included in the preliminary screening analyses. Mahalanobis distance (D^2^) was evaluated against a chi-square distribution with 5 degrees of freedom, corresponding to the number of variables included in the analysis. A criterion of *p* < 0.001 was used to identify multivariate outliers, yielding a critical value of χ^2^(5) = 20.515. Based on this criterion, 13 participants with D^2^ values exceeding 20.515 were identified as multivariate outliers and excluded from subsequent analyses. Following the exclusion of these participants, the final analytic sample consisted of 608 participants (Tabachnick & Fidell, 2019; Hair et al., 2019).

### 3.3. Measures

#### 3.3.1. Personal Information Form

Demographic information regarding the participating children and their parents was collected using a Personal Information Form developed by the researchers. The form included questions on the child’s age and gender, together with parental demographic characteristics, including age, educational attainment, and employment status. In addition, information on the family’s socioeconomic status was obtained through this form.

#### 3.3.2. Adult Attachment Styles Scale

Adult attachment styles were assessed using the Adult Attachment Styles Scale, which was originally developed through the successive work of Hazan and Shaver (1987) and. The Turkish version of the instrument was adapted and psychometrically validated by Kesebir et al. (2012). As part of the adaptation process, several items were reworded and divided into separate statements to improve clarity, resulting in an 18-item version of the scale. The instrument comprises three six-item subscales representing secure, avoidant, and anxious/ambivalent attachment styles. Individuals’ dominant attachment style is identified according to the subscale on which they obtain the highest score. In the Turkish validation study, the internal consistency coefficients (Cronbach’s α) were reported as 0.72 for the secure attachment subscale, 0.82 for the avoidant attachment subscale, and 0.85 for the anxious/ambivalent attachment subscale (Kesebir et al., 2012). For the purposes of the present study, only the avoidant and anxious/ambivalent attachment subscales were included in the analyses.

#### 3.3.3. Social Competence and Behavior Evaluation Scale–30

Social Competence and Behavior Evaluation Scale–30 (SCBE–30) was developed by P. J. LaFreniere and Dumas (1996) to evaluate social competence and behavioral adjustment in preschool children between 3 and 6 years of age. The instrument consists of three subscales: Social Competence, Anxiety–Withdrawal, and Anger–Aggression. The Turkish version of the SCBE-30 was adapted and validated by Çorapçı et al. (2010), who reported satisfactory internal consistency coefficients (Cronbach’s α) of 0.84 for the Social Competence subscale, 0.88 for the Anxiety–Withdrawal subscale, and 0.87 for the Anger–Aggression subscale. In the present study, analyses were conducted using only the 10-item Anxiety–Withdrawal subscale. The Anxiety–Withdrawal subscale of the SCBE-30 was used to assess anxiety-related behaviors and behaviors characterized by social withdrawal.

#### 3.3.4. Technoference in Parent–Child Relationships Scale

Technoference in Parent–Child Relationships Scale (TPCRS) was originally developed by McDaniel and Radesky (2018) to measure the extent to which technology use disrupts interactions between parents and their children. The instrument was later translated, culturally adapted, and psychometrically validated for use in the Turkish context by Özcan and Tezel-Şahin (2025). The Turkish validation study demonstrated satisfactory internal consistency, with a Cronbach’s alpha coefficient of 0.70. Construct validity was further supported by favorable confirmatory factor analysis fit indices (χ^2^/df = 1.828, GFI = 0.97, NFI = 0.96, CFI = 0.98, AGFI = 0.96, SRMR = 0.056, and RMSEA = 0.046). The TPCRS is designed for completion by either mothers or fathers. Higher total scores indicate more frequent technology-related disruptions during parent–child interactions, reflecting greater levels of technoference within the parent–child relationship.

#### 3.3.5. Strengths and Difficulties Questionnaire

The Strengths and Difficulties Questionnaire (SDQ) was introduced by Goodman (1997) as a screening instrument for evaluating emotional and behavioral functioning in children and adolescents aged 4 to 16 years. The questionnaire contains 25 items and is available in both parent- and teacher-report formats. Unlike many measures that focus solely on behavioral difficulties, the SDQ also assesses children’s strengths by examining domains such as prosocial behavior, emotional symptoms, conduct problems, hyperactivity, and peer relationships. Each of the five subscales is scored separately, allowing for the assessment of distinct aspects of psychological functioning. The Turkish version of the SDQ was first adapted by Güvenir et al. (2008), who reported a Cronbach’s alpha coefficient of 0.65 for the total scale after excluding the peer problems subscale. Subsequently, Yalin (2008) reported an internal consistency coefficient of 0.70 using the same scoring approach. In the present study, only the Prosocial Behavior subscale was included in the analyses. The decision to use the Prosocial Behavior subscale of the SDQ rather than the Social Competence subscale of the SCBE-30 to assess prosocial behavior was made in line with the conceptual focus of the study. The Prosocial Behavior subscale of the SDQ specifically assesses positive behaviors directed toward others, such as helping, sharing, and cooperating, whereas the Social Competence subscale of the SCBE-30 represents a broader domain of social functioning. Therefore, in the present study, prosocial behavior was conceptualized as a specific behavioral construct.

### 3.4. Data Analysis

Prior to the primary analyses, the distributional properties of the data were examined to verify the assumptions required for parametric statistical analyses. Pearson product–moment correlation coefficients were then calculated to investigate the associations among the study variables. To evaluate the internal consistency of the measurement instruments, Cronbach’s α, Guttman’s λ6, and McDonald’s ω reliability coefficients were computed. The hypothesized model was tested using the two-step structural equation modeling (SEM) procedure recommended by Anderson and Gerbing (1988). First, a confirmatory factor analysis (CFA) was conducted to evaluate the adequacy of the measurement model. After establishing satisfactory measurement properties, the structural model was estimated to examine the hypothesized relationships among the latent constructs. Model adequacy for both the measurement and structural models was evaluated using multiple goodness-of-fit indices. The fit statistics were interpreted according to the criteria proposed by Kline (2015), whereby RMSEA and SRMR values below 0.08, together with CFI, NFI, TLI, and GFI values exceeding 0.90, were considered indicative of acceptable model fit. Child gender and age were included as covariates because they may be potentially associated with children’s social-emotional and behavioral functioning and, therefore, may account for some variance in the child outcome variable independently of the key variables included in the proposed model. Their inclusion was intended to account for potential variation in the outcome associated with child-level demographic characteristics rather than to statistically control for all possible confounding variables. All statistical analyses were performed using IBM SPSS Statistics 22.0, IBM AMOS 24.0, and JASP 0.17.1.

## 4. Results

### 4.1. Preliminary Analysis

Table 1 presents the descriptive statistics, reliability coefficients (Cronbach’s α, McDonald’s ω, and Guttman’s λ6), skewness and kurtosis values, as well as the bivariate correlations among the study variables. Examination of the distributional characteristics indicated that all variables met the assumption of univariate normality, with skewness values ranging from −0.37 to 1.33 and kurtosis values ranging from −0.21 to 1.88. These values fall within the commonly accepted ±2 threshold, indicating that the data adequately met the assumption of normality with respect to distributional characteristics (D. George & Mallery, 2010). The findings revealed that anxious/ambivalent attachment was positively associated with avoidant attachment (r = 0.39), anxiety–withdrawal (r = 0.43), and technoference (r = 0.34), while it was negatively associated with prosocial behavior (r = −0.27). Anxiety–withdrawal demonstrated positive correlations with avoidant attachment (r = 0.12) and technoference (r = 0.45), and a negative correlation with prosocial behavior (r = −0.53). Furthermore, technoference was negatively associated with prosocial behavior (r = −0.33). In addition, the reliability coefficients calculated for all measurement instruments indicated satisfactory internal consistency (α ≥ 0.70, ω ≥ 0.72, and λ6 ≥ 0.72).

### 4.2. Measurement Model

A measurement model consisting of five latent variables (anxious attachment, avoidant attachment, technoference, anxiety–withdrawal, and prosocial behavior) and 10 observed indicators was examined. The measurement model was evaluated using confirmatory factor analysis (CFA). The results demonstrated that the proposed model showed an acceptable fit to the observed data (χ^2^ [25, N = 608] = 70.358, *p* < 0.001; χ^2^/df = 2.814; GFI = 0.977; CFI = 0.983; AGFI = 0.949; NFI = 0.975; IFI = 0.984; SRMR = 0.043; RMSEA = 0.055). The standardized factor loadings of the observed indicators on their respective latent variables ranged from 0.54 to 0.93, and all factor loadings were statistically significant. These findings indicate that the observed indicators adequately represented their underlying latent constructs, supporting the validity and adequacy of the measurement model.

### 4.3. Structural Model

Following confirmation that the measurement model demonstrated an acceptable level of fit, the structural model analyses were conducted. In line with the recommendations of Kline (2015), Structural Equation Modeling (SEM) analyses were performed using AMOS Graphics following a two-step approach. The bootstrap procedure was employed to assess the statistical significance of the indirect effects. Several goodness-of-fit indices were examined to evaluate the adequacy of the structural model. To represent latent constructs through observed indicators, the item parceling approach was adopted. This strategy was used to reduce the number of observed variables, facilitate the modeling of unidimensional constructs, improve distributional properties, and enhance measurement reliability (Bandalos, 2008; Little et al., 2002). Before constructing the item parcels, the factor structure of the items for each construct was examined separately at the item level using confirmatory factor analysis (CFA). This preliminary evaluation was conducted to assess the measurement structure of the constructs before individual items were combined into parcels and to identify potential item-level issues that might otherwise be obscured by the parceling procedure. The item-level CFA results for anxious-ambivalent attachment, avoidant attachment, technoference, prosocial behavior, and anxiety–withdrawal were evaluated based on the model fit indices and standardized factor loadings. Overall, these analyses provided evidence supporting the intended factor structures. Following the item-level assessment, two item parcels were created for each latent construct. The items were systematically distributed across the two parcels according to their item numbers, with odd-numbered items assigned to one parcel and even-numbered items assigned to the other. This systematic allocation procedure was applied consistently across all relevant constructs. The resulting item parcels were subsequently used as observed indicators of the corresponding latent constructs in the measurement and structural models. In addition, child gender and age were included in the models as control variables. To evaluate the hypothesized structural associations, two separate models were tested.

**Figure 1:**
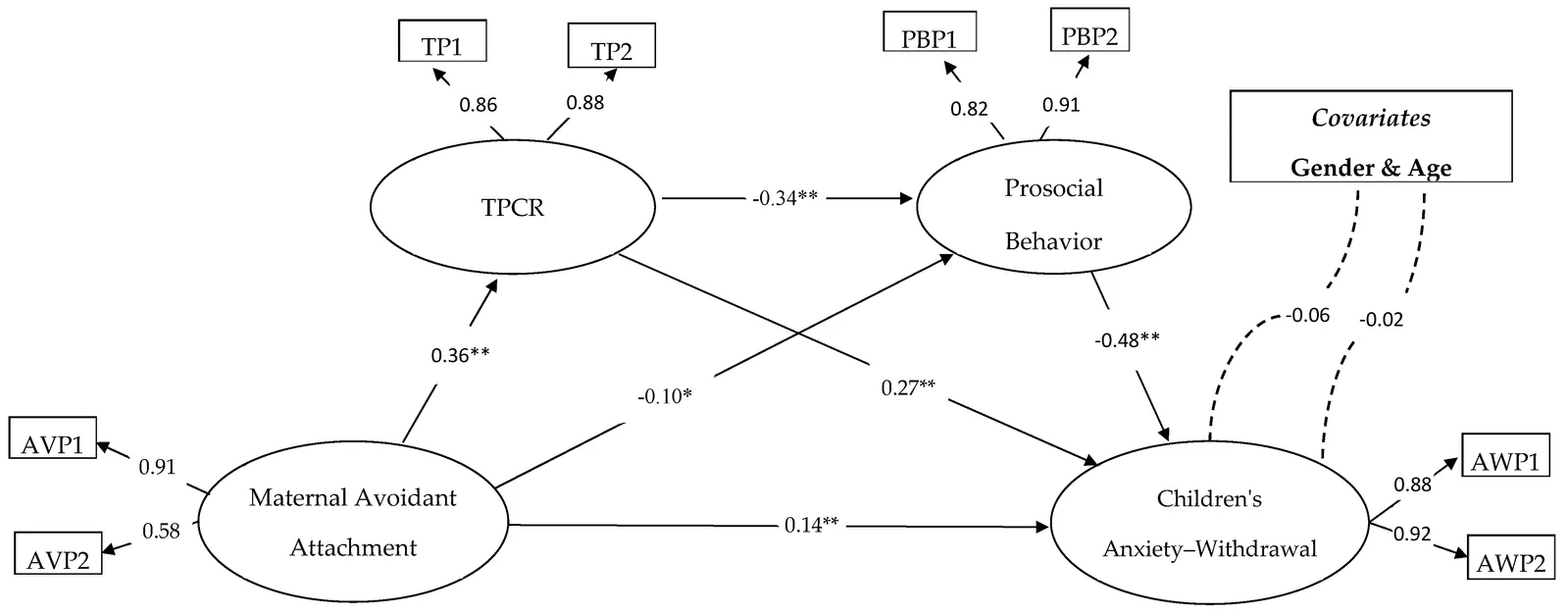
The Mediating Roles of Technoference and Prosocial Behavior in the Associations Between Maternal Avoidant Attachment Styles and Anxiety–Withdrawal Levels in Preschool-Aged Children. Note. * *p* < 0.05, ** *p* < 0.01, TPCR: technoference in parent–child relationships, TP: parcel of technoference, PBP: parcel of prosocial behavior, AWP: parcel of children’s anxiety–withdrawal, AVP: parcel of maternal avoidant attachment.

Model 1 (Avoidant Attachment Model). First, Model 1 was tested to examine the structural associations among maternal avoidant attachment, parental technoference, prosocial behavior, and anxiety–withdrawal. A partial mediation model including all hypothesized direct and indirect paths was initially tested. The results indicated that all specified structural paths were statistically significant and that the model demonstrated good fit to the data (χ^2^[29, N = 608] = 31.267, *p* = 0.353; χ^2^/df = 1.078; GFI = 0.990; AGFI = 0.981; CFI = 0.998; NFI = 0.987; IFI = 0.999; SRMR = 0.025; RMSEA = 0.013; AIC = 83.267; ECVI = 0.137). Subsequently, a full mediation model was tested by constraining the direct path from maternal avoidant attachment to anxiety–withdrawal to zero. The results indicated that the full mediation model also demonstrated good fit to the data (χ^2^[30, N = 608] = 42.370, *p* = 0.067; χ^2^/df = 1.412; GFI = 0.986; AGFI = 0.975; CFI = 0.995; NFI = 0.982; IFI = 0.995; SRMR = 0.031; RMSEA = 0.026; AIC = 92.370; ECVI = 0.152). All structural paths specified in this model were also statistically significant. To determine the more appropriate model, the AIC and ECVI values were compared, with lower values indicating a better balance between model fit and parsimony (Browne & Cudeck, 1992). In addition, a chi-square difference test was conducted to compare the partial and full mediation models. The chi-square difference test indicated that the partial mediation model provided a statistically significant improvement in model fit relative to the full mediation model, Δχ^2^(1) = 11.310, *p* < 0.001. Consistent with this result, the partial mediation model yielded lower AIC and ECVI values than the full mediation model. Accordingly, the partial mediation model was retained as the final model. The standardized path coefficients for the final partial mediation model are presented in Figure 1.

**Figure 2:**
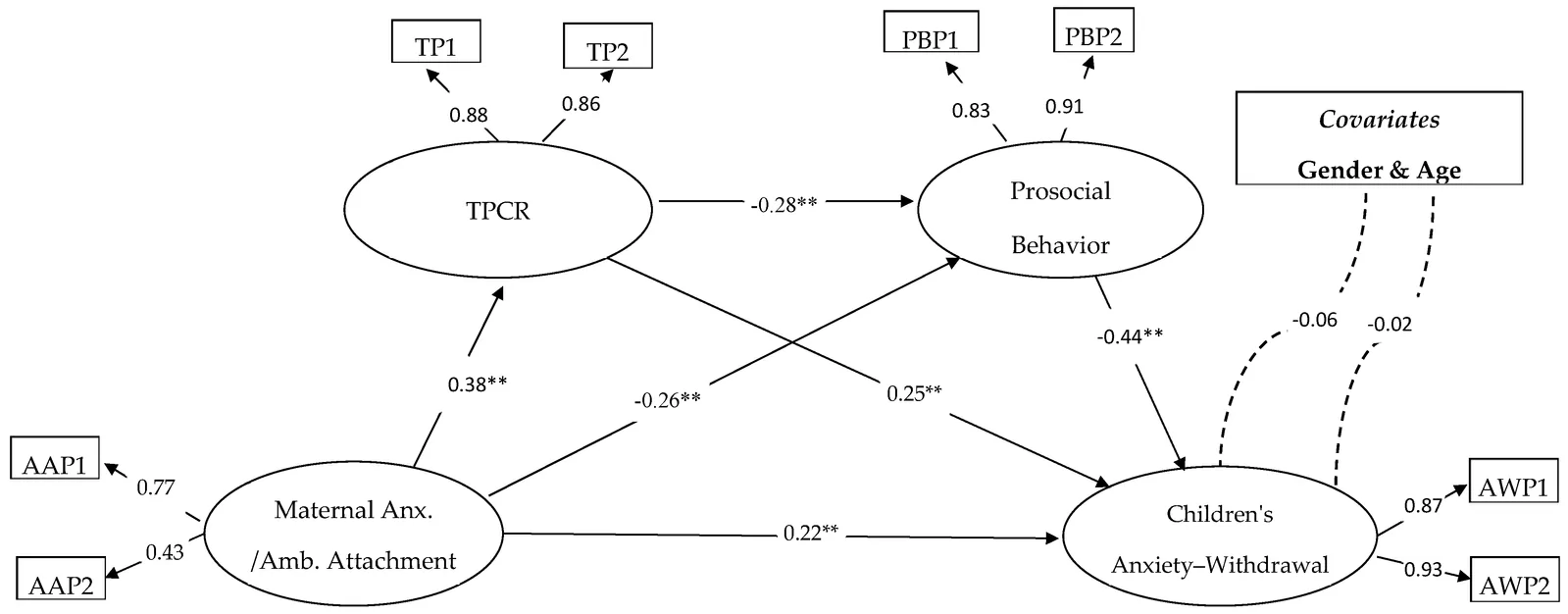
The Mediating Roles of Technoference and Prosocial Behavior in the Associations Between Maternal Anxious/Ambivalent Attachment Styles and Anxiety–Withdrawal Levels in Preschool-Aged Children. Note. ** *p* < 0.01, Maternal Anx./Amb. Attachment: anxious/ambivalent attachment, TPCR: technoference in parent–child relationships, TP: parcel of technoference, PBP: parcel of prosocial behavior, AWP: parcel of children’s anxiety–withdrawal, AAP: parcel of maternal anxious/ambivalent attachment.

Model 2 (Anxious/Ambivalent Attachment Model). In the second stage, Model 2 was tested to examine the structural associations among maternal anxious/ambivalent attachment, parental technoference, prosocial behavior, and anxiety–withdrawal. A partial mediation model including all hypothesized direct and indirect paths was initially tested. The results indicated that all specified structural paths were statistically significant and that the model demonstrated good fit to the data (χ^2^[14, N = 608] = 15.714, *p* = 0.331; χ^2^/df = 1.122; GFI = 0.994; AGFI = 0.984; CFI = 0.998; NFI = 0.993; IFI = 0.998; SRMR = 0.017; RMSEA = 0.014; AIC = 59.714; ECVI = 0.098). Subsequently, a full mediation model was tested by constraining the direct path from maternal anxious/ambivalent attachment to anxiety–withdrawal to zero. The results indicated that the full mediation model also demonstrated good fit to the data (χ^2^[30, N = 608] = 48.545, *p* = 0.017; χ^2^/df = 1.618; GFI = 0.985; AGFI = 0.972; CFI = 0.992; NFI = 0.978; IFI = 0.992; SRMR = 0.036; RMSEA = 0.032; AIC = 98.545; ECVI = 0.162). All structural paths specified in this model were also statistically significant. To evaluate the relative fit of the competing models, AIC and ECVI values were compared, with lower values indicating better model fit while accounting for model complexity (Browne & Cudeck, 1992). In addition, a chi-square difference test was conducted to compare the partial and full mediation models. The results indicated that the partial mediation model provided a statistically significant improvement in model fit relative to the full mediation model, Δχ^2^(1) = 20.638, *p* < 0.001. Taken together, the lower AIC and ECVI values for the partial mediation model and the significant chi-square difference test supported retaining the partial mediation model as the final model. The standardized path coefficients for the final model are presented in Figure 2.

In addition to the structural equation modeling analyses, a bootstrap procedure with 5000 resamples was conducted to estimate 95% confidence intervals for the indirect effects. In bootstrap-based mediation analyses, an indirect effect is considered statistically supported when its 95% confidence interval does not include zero (Gürbüz, 2019). In the present study, two models (Model 1 and Model 2) were examined to investigate the indirect associations involving maternal insecure attachment. As presented in Table 2, all direct associations among the variables in Model 1 were statistically significant (*p* < 0.05 or *p* < 0.01). The bootstrap analysis further indicated a statistically significant indirect association between maternal avoidant attachment and children’s anxiety–withdrawal through parental technoference (B = 0.321, 95% bootstrap CI [0.191, 0.506], β = 0.097). In addition, prosocial behavior was found to significantly mediate the association between maternal insecure attachment and children’s anxiety–withdrawal (B = 0.165, 95% bootstrap CI [0.006, 0.369], β = 0.048). Finally, consistent with the primary hypothesis of Model 1, the bootstrap analysis indicated a statistically significant serial indirect association between maternal avoidant attachment and children’s anxiety–withdrawal through parental technoference and prosocial behavior in sequence (B = 0.195, 95% bootstrap CI [0.118, 0.311], β = 0.058). These findings provide empirical support for a serial indirect association model in which higher levels of maternal avoidant attachment are associated with greater parental technoference, which is, in turn, associated with children’s prosocial behavior and subsequently with their anxiety–withdrawal levels during the preschool period. In addition, neither child gender nor child age, which were included in the analyses as covariates, was significantly associated with the dependent variable.

In addition, as presented in Table 3, all direct associations among the variables in Model 2 were statistically significant (*p* < 0.01). The bootstrap analysis further indicated a statistically significant indirect association between maternal anxious/ambivalent attachment and children’s anxiety–withdrawal through parental technoference (B = 0.401, 95% bootstrap CI [0.224, 0.671], β = 0.095). In addition, prosocial behavior was found to significantly mediate the association between maternal insecure attachment and children’s anxiety–withdrawal (B = 0.478, 95% bootstrap CI [0.238, 0.852], β = 0.114). Finally, consistent with the primary hypothesis of Model 2, the bootstrap analysis indicated a statistically significant serial indirect association between maternal anxious/ambivalent attachment and children’s anxiety–withdrawal through parental technoference and prosocial behavior in sequence (B = 0.199, 95% bootstrap CI [0.108, 0.341], β = 0.046). These findings provide empirical support for a serial indirect association model in which higher levels of maternal anxious/ambivalent attachment are associated with greater parental technoference, which is, in turn, associated with children’s prosocial behavior and subsequently with their anxiety–withdrawal levels during the preschool period. In addition, neither child gender nor child age, which were included in the analyses as covariates, was significantly associated with the dependent variable.

## 5. Discussion

The present study investigated a serial mediation model to examine whether technoference and prosocial behavior statistically accounted for the association between mothers’ avoidant and anxious/ambivalent attachment and anxiety–withdrawal in preschool-aged children. By examining these associations simultaneously, the study offers a broader perspective on how maternal attachment orientations relate to children’s social, emotional, and psychological adjustment. The main objective was therefore to evaluate the mediating functions of technoference and prosocial behavior in the relationship between maternal avoidant and anxious/ambivalent attachment and preschoolers’ anxiety–withdrawal. The results indicated that the associations between maternal avoidant and anxious/ambivalent attachment and children’s anxiety were statistically accounted for by the serial mediating roles of technoference and prosocial behavior. Given that mothers’ attachment representations have been associated with the quality of parent–child interactions and may be relevant to children’s long-term emotional and psychological adjustment (Rost et al., 2024), these findings may have implications for intervention efforts. Specifically, they suggest that strategies aimed at reducing technoference, strengthening parent–child relationships, and fostering children’s prosocial development may support children’s mental well-being and be associated with lower levels of emotional and behavioral difficulties. The individual pathways underlying this model are discussed in the following sections.

Results from the first research question indicated that maternal avoidant and anxious/ambivalent attachment was significantly positively associated with anxiety–withdrawal levels in preschool-aged children. The literature provides evidence of an association between parental insecure attachment and children’s anxiety levels (Cooke et al., 2019; Rost et al., 2024). This association suggests that insecure maternal attachment patterns may be linked to children’s social, emotional, and psychological adjustment. Attachment style has been associated with individuals’ attitudes and behaviors in both personal and close relationships (Ebrahimi et al., 2017). In the context of the mother–child relationship, insecure attachment patterns may therefore be relevant to children’s social and psychological adjustment. Consistent with existing theoretical and empirical evidence, the positive associations observed between maternal insecure attachment and children’s anxiety–withdrawal are in line with theoretically expected patterns.

The second finding of this study demonstrated that technoference significantly mediated the association between mothers’ avoidant and anxious/ambivalent attachment and anxiety–withdrawal in their preschool-aged children (RQ2). This result can be interpreted within the framework of attachment theory, which suggests that attachment orientations are associated with individuals’ capacity for stress management, emotion regulation, and interpersonal relationships, which may, in turn, be reflected in their technology-related behaviors (Mikulincer & Shaver, 2016). Previous research has consistently indicated that insecure attachment, particularly anxious and avoidant attachment patterns, is associated with greater susceptibility to problematic internet use and excessive social media engagement (Huang et al., 2025; Niu et al., 2023). Although these studies have primarily examined technology-related behaviors in broader or non-preschool populations, their findings provide a relevant conceptual context for considering technology-related behaviors among parents of preschool-aged children. Young (1998) further argued that individuals with anxious attachment may increasingly rely on online environments to obtain the reassurance and approval they struggle to achieve in face-to-face relationships. Empirical findings also indicate that individuals characterized by insecure attachment orientations spend more time using digital technologies and exhibit a higher likelihood of developing technology-related addictive behaviors (Erözkan, 2011; Monacis et al., 2017). When these patterns extend to parenting, excessive digital device use may interfere with parent–child interactions through technoference. Frequent interruptions from smartphones and other digital media may be associated with reduced verbal and nonverbal communication and with differences in the frequency of responsive and emotionally meaningful interactions between parents and children (J. Radesky et al., 2015). Such disruptions may gradually weaken the emotional bond within the parent–child relationship, increasing children’s vulnerability to emotional and behavioral difficulties, including both internalizing and externalizing problems (Di Maggio & Zappulla, 2014; Xie & Xie, 2020). Evidence from previous studies further supports this explanation. For example, Kurban (2023) reported that stronger parent–child relationship quality was associated with lower levels of problematic technology use among children. In contrast, greater problematic technology use was linked to higher levels of childhood anxiety–withdrawal. More recent evidence suggests that the association between insecure attachment and technology use extends beyond observable behavior to underlying neurobiological processes. Eichenberg et al. (2024) demonstrated that individuals with anxious attachment showed greater activation of neural reward-processing systems in response to social media notifications, suggesting an increased sensitivity to digitally mediated social rewards and affiliation cues. Taken together, these findings suggest that mothers with insecure attachment orientations may report greater reliance on digital platforms, such as social media and the internet, for emotional reassurance, acceptance, or interpersonal connection. Greater reliance on these digital environments may be associated with differences in the frequency and quality of parent–child interactions, with implications for emotional attunement and sensitive caregiving. These interactional disruptions may be associated with higher levels of internalizing difficulties, particularly anxiety–withdrawal, among children. Accordingly, the present findings underscore the importance of intervention strategies that promote parents’ awareness of technoference, encourage healthier technology-use habits, and strengthen the quality and consistency of parent–child interactions as central components of family-based prevention and intervention programs.

The findings further indicated that prosocial behavior significantly mediated the relationship between mothers’ avoidant and anxious/ambivalent attachment and anxiety–withdrawal in preschool-aged children (RQ3). More specifically, higher levels of maternal insecure (avoidant and anxious/ambivalent) attachment were associated with lower levels of children’s prosocial behavior, which, in turn, was linked to elevated anxiety–withdrawal. Although no previous study has simultaneously investigated maternal insecure attachment, prosocial behavior, and preschool children’s anxiety–withdrawal within a single analytical framework, substantial evidence supports the associations among these constructs. Earlier research has consistently shown that maternal attachment insecurity is related to children’s emotional adjustment. For example, Costa and Weems (2005) found that mothers’ attachment-related anxiety was associated with higher anxiety in their children. Likewise, Gross et al. (2017) reported a close relationship between the quality of parent–child attachment and children’s prosocial behavior. Expanding on these findings, Santelices et al. (2024) reported that parental reflective functioning and depressive symptoms statistically accounted for the association between maternal insecure attachment and children’s socio-emotional development. In addition, Zhang et al. (2023) identified a negative relationship between prosocial behavior and symptoms of depression and anxiety–withdrawal, suggesting that children who display greater prosocial tendencies tend to experience fewer internalizing difficulties. Prosocial behavior is widely regarded as a socially valued characteristic that is reinforced through positive interpersonal feedback and social approval, both of which have been associated with psychological well-being (Hay, 1994). Beyond its social value, previous studies have suggested that engagement in prosocial behaviors is associated with lower levels of anxiety and other forms of negative emotionality (Fujiwara, 2007; Grant & Sonnentag, 2010). From this perspective, prosocial behavior may represent one potential factor associated with the relationship between maternal insecure attachment (avoidant and anxious/ambivalent) and children’s emotional adjustment. Higher levels of prosocial functioning may also be associated with broader social competencies, which, in turn, have been linked to more effective emotion regulation and self-regulation and to lower levels of anxiety and aggressive behavior (P. LaFreniere et al., 2002). Evidence from intervention research broadly supports this interpretation. Programs designed to enhance children’s prosocial behaviors have been associated with improvements in social and emotional functioning (Domitrovich et al., 2007; Pahl & Barrett, 2007). Collectively, the present findings suggest that childhood anxiety–withdrawal may be better understood within a broader developmental framework that encompasses both family attachment processes and children’s social competencies. Accordingly, interventions targeting early childhood anxiety–withdrawal may benefit from considering both parent–child attachment relationships and children’s prosocial skills as potentially relevant intervention targets.

The final finding of this study indicates that parental technoference and children’s prosocial behavior serially mediate the association between maternal avoidant and anxious/ambivalent attachment and anxiety–withdrawal in preschool children (RQ4). This finding supports the study’s primary hypothesis. This pattern suggests that mothers with insecure attachment orientations may be more likely to experience digital technologies as interfering with interactions with their children. As a result, opportunities for children to develop and practice prosocial behaviors may be less frequent, which may, in turn, be associated with higher levels of anxiety–withdrawal. This interpretation is broadly consistent with previous evidence indicating that individuals characterized by avoidant or anxious/ambivalent attachment are more likely to report problematic technology use and excessive engagement with social media (Huang et al., 2025; Musetti et al., 2022). Likewise, studies have reported that children whose parents use digital technologies more intensively tend to display lower levels of positive social behavior (Komanchuk et al., 2023; Toledo-Vargas et al., 2025). In addition, lower levels of prosocial competence have been associated with higher levels of anxiety and other internalizing symptoms during childhood (Valdes et al., 2025; Zhang et al., 2023). Collectively, these findings are consistent with the possibility that technology-related disruptions in parent–child interactions may be associated with less favorable social development, which, in turn, may relate to greater vulnerability to anxiety–withdrawal. More broadly, the present findings suggest that maternal avoidant and anxious/ambivalent attachment may represent one factor associated with childhood anxiety–withdrawal. They also indicate that technoference may be indirectly associated with the association between maternal attachment insecurity and children’s emotional adjustment. Furthermore, the results suggest that maternal insecure attachment may be associated with children’s anxiety–withdrawal through a serial pathway involving parental technoference and lower levels of children’s prosocial behavior. Taken together, these findings suggest that childhood anxiety may be better understood within a multidimensional developmental framework that incorporates parental attachment representations, technology-related disruptions in parent–child interactions, and children’s social competencies. From a practical perspective, interventions that encourage mindful parental technology use, support secure and responsive parent–child relationships, and promote children’s prosocial skills may help foster children’s emotional well-being. Nevertheless, longitudinal and experimental research is needed to clarify the temporal and potentially causal mechanisms underlying these associations and to inform the development of evidence-based family-centered prevention and intervention programs.

## 6. Limitations and Future Research

Although the findings are important and meaningful, they should be interpreted in the context of several methodological limitations. First, although using multiple informants is a methodological strength, informant-specific bias cannot be completely ruled out. Mothers assessed maternal attachment and parental technoference, whereas teachers assessed children’s anxiety–withdrawal and prosocial behaviors. However, observational data could have provided additional objective evidence of parent–child interactions and children’s behaviors. Moreover, assessing attachment and technoference by the same maternal informant and anxiety–withdrawal and prosocial behavior by the same teacher may have introduced some degree of common informant bias. In addition, the study’s cross-sectional design allows examination of relationships among variables only within a specific time frame and limits causal inference. Considering that the associations between parental attachment styles and children’s anxiety–withdrawal may vary across developmental contexts, future longitudinal and experimental studies could provide a more comprehensive understanding of the nature and direction of these associations. Another important limitation is that the study used only quantitative methods. While quantitative data are useful for identifying relationships among variables, they may not fully capture the complex underlying dynamics of psychological and relational processes. Therefore, future qualitative or mixed-methods studies are expected to provide a more in-depth and multidimensional understanding of parental attachment styles and related psychosocial processes. Furthermore, convenience sampling is another methodological limitation that may restrict the generalizability of the findings. Samples drawn from readily accessible participants may not adequately represent the target population, thereby increasing the risk of sampling bias. Accordingly, future research should employ alternative sampling strategies, such as random or probability sampling methods. Additionally, when interpreting the observed associations, it should be acknowledged that unmeasured maternal and child characteristics, such as maternal psychopathology, parenting stress, and child temperament, may have influenced these associations. Finally, the present study’s sample was limited to children aged 60–72 months attending independent preschools in the central districts of Denizli province and their parents. This restriction limits the generalizability of the findings to different age groups, educational settings, and cultural contexts. Therefore, testing the proposed model in more diverse socio-cultural contexts and on more heterogeneous samples is expected to strengthen the external validity and theoretical generalizability of the findings.

## 7. Implications

This study makes several important theoretical contributions to the literature; a comprehensive model is proposed that examines the associations between mothers’ avoidant and anxious/ambivalent attachment and preschool children’s anxiety–withdrawal through the serially mediating roles of parental technoference and prosocial behavior. The findings suggest that attachment theory may be extended beyond interpersonal relationships to encompass parenting practices in the digital age. In particular, the observed indirect association involving parental technoference provides a potential framework for understanding the link between attachment-related internal working models and technology-related parenting behaviors, thereby contributing to the integration of research on attachment, digital parenting, and child mental health. From a practical perspective, the findings may help identify potential areas for future prevention and intervention research concerning the quality of parent–child interactions and parents’ technology use. For example, increasing parents’ awareness of their digital device use, particularly among parents with insecure attachment orientations, and examining whether educational approaches targeting parental technoference and parental sensitivity are feasible and effective may represent promising directions for future research. Similarly, the observed finding regarding prosocial behavior suggests that children’s social competence may be a promising area for future prevention research. Future intervention studies could examine school-based social–emotional learning programs that promote prosocial behavior to determine whether strengthening children’s social competence is associated with changes in anxiety–withdrawal. However, given the cross-sectional nature of the data, the present findings should not be interpreted as evidence that promoting prosocial behavior would necessarily be associated with lower levels of children’s anxiety or serve a protective function. From a policy and educational perspective, the findings may support future discussions regarding family- and school-based approaches to mental health promotion in early childhood. Parent involvement initiatives, digital awareness programs, and attachment-informed parenting education implemented within preschool settings may warrant further investigation as potential components of preventive programs. Similarly, future intervention research could examine whether approaches that consider children’s developmental needs alongside parents’ psychological characteristics and digital behaviors provide additional benefits. These implications should nevertheless be viewed as directions for future research rather than recommendations for current intervention practice. Finally, the findings underscore the importance of conceptualizing childhood anxiety–withdrawal as a multidimensional phenomenon that may be associated with individual, relational, and environmental factors. Considering maternal attachment style, technology-related disruptions in parent–child interactions, and children’s social behavior together may provide a more comprehensive framework for understanding factors associated with children’s anxiety–withdrawal. Accordingly, future research should employ longitudinal and experimental designs to determine whether these variables represent modifiable intervention targets and to examine whether changes in these factors are associated with subsequent changes in children’s anxiety–withdrawal. Such research may provide a basis for developing and evaluating evidence-based prevention and intervention strategies.

## 8. Conclusions

This study examines whether parental technoference and children’s prosocial behavior sequentially account for the association between mothers’ avoidant and anxious/ambivalent attachment and preschool children’s anxiety–withdrawal. The findings highlight parents’ technology use, parent–child interaction processes, and children’s prosocial behavior as areas warranting further investigation. Accordingly, these findings may provide a basis for future longitudinal and intervention research examining whether parents’ awareness of their technology use, the quality of parent–child interactions, and children’s prosocial behavior are modifiable factors associated with anxiety–withdrawal. Future studies should determine whether interventions targeting these areas are feasible and effective and whether changes in these factors are associated with subsequent changes in children’s anxiety–withdrawal. Until such evidence is available, these variables should be considered potential targets for future prevention research rather than established intervention mechanisms for reducing childhood anxiety–withdrawal.

## Figures and Tables

**Table 1:** Descriptive statistics and correlation coefficients among variables.

	Descriptive Statistics and Reliabilities							Correlations			
Variables	Mean	SD	Skewness	Kurtosis	α	ω	λ6	1	2	3	4
1. Anxious/Ambivalent Attachment	14.87	4.19	−0.010	−0.215	0.76	0.77	0.78	-			
2. Avoidant Attachment	17.34	3.79	−0.370	0.983	0.70	0.72	0.72	0.39 **	-		
3. Anxiety–Withdrawal	21.16	8.58	0.850	0.350	0.85	0.88	0.87	0.43 **	0.12 **	-	
4. TPCR	13.61	5.97	1.33	1.88	0.82	0.83	0.83	0.34 **	0.18 **	0.45 **	-
5. Prosocial Behavior	10.34	4.09	0.533	−0.117	0.82	0.84	0.84	−0.27 **	−0.11 *	−0.53 **	−0.33 **

Note. * *p* < 0.05; ** *p* < 0.01; TPCR: Technoference in parent–child relationships.

**Table 2:** Examination of the direct and indirect effects in model 1.

				Bootstrap 95% CI		
Model-1 Pathways	B	β	SE	Lower	Upper	*p*
Direct effects						
Avoidant attachment → Anxiety–withdrawal	0.446	0.136	0.045	0.050	0.228	<0.002
Avoidant attachment → Technoference	0.802	0.363	0.051	0.258	0.456	<0.001
Avoidant attachment → Prosocial	−0.180	−0.105	0.052	−0.206	−0.004	<0.050
Technoference → Prosocial	−0.264	−0.341	0.049	−0.437	−0.242	<0.001
Technoference → Anxiety–withdrawal	0.401	0.270	0.051	0.172	0.372	<0.001
Prosocial → Anxiety–withdrawal	−0.920	−0.480	0.046	−0.567	−0.384	<0.002
Indirect effects						
Avoidant attachment → Technoference → Anxiety–withdrawal	0.321	0.097	0.080	0.191	0.506	<0.001
Avoidant attachment → Prosocial → Anxiety–withdrawal	0.165	0.048	0.092	0.006	0.369	<0.050
Avoidant attachment → Technoference → Prosocial → Anxiety–withdrawal	0.195	0.058	0.048	0.118	0.311	<0.001
Total effect	1.127	0.343	0.050	0.246	0.444	<0.001
Total indirect effect	0.681	0.203	0.036	0.142	0.283	<0.001

Note: CI confidence interval; B unstandardized effect; β = standardized effect; Indirect effects were evaluated using bootstrap 95% confidence intervals; effects were considered statistically significant when the confidence interval did not include zero. The 95% bootstrap confidence intervals (lower and upper bounds) for the indirect effects were estimated on the unstandardized scale using the AMOS User-Defined Estimands procedure.

**Table 3:** Examination of the direct and indirect effects in model 2.

				Bootstrap 95% CI		
Model-2 Pathways	B	β	SE	Lower	Upper	*p*
Direct effects						
Anx./Amb. attachment → Anxiety–withdrawal	0.961	0.224	0.062	0.114	0.359	<0.001
Anx./Amb. attachment → Technoference	1.114	0.375	0.066	0.242	0.497	<0.001
Anx./Amb. attachment → Prosocial	−0.575	−0.165	0.060	−0.385	−0.145	<0.001
Technoference → Prosocial	−0.215	−0.284	0.053	−0.383	−0.176	<0.001
Technoference → Anxiety–withdrawal	0.360	0.250	0.052	0.147	0.353	<0.001
Prosocial → Anxiety–withdrawal	−0.830	−0.457	0.049	−0.529	−0.337	<0.001
Indirect effects						
Anx./Amb. attachment → Technoference → Anxiety–withdrawal	0.401	0.095	0.112	0.224	0.671	<0.001
Anx./Amb. attachment → Prosocial → Anxiety–withdrawal	0.478	0.114	0.154	0.238	0.852	<0.001
Anx./Amb. attachment → Technoference → Prosocial → Anxiety–withdrawal	0.199	0.046	0.058	0.108	0.341	<0.001
Total effect	2.039	0.476	0.064	0.349	0.603	<0.001
Total indirect effect	1.078	0.255	0.037	0.187	0.333	<0.001

Note: CI confidence interval; Anx./Amb. anxious/ambivalent; B unstandardized effect; β = standardized effect; Indirect effects were evaluated using bootstrap 95% confidence intervals; effects were considered statistically significant when the confidence interval did not include zero. The 95% bootstrap confidence intervals (lower and upper bounds) for the indirect effects were estimated on the unstandardized scale using the AMOS User-Defined Estimands procedure.

## Data Availability

The datasets created and/or analyzed as part of this study are not publicly available because they contain individual-level data collected from preschool-aged children and their parents, and unrestricted public access could jeopardize participants’ privacy and confidentiality. However, the data may be made available by the corresponding author upon reasonable request and subject to appropriate ethical and confidentiality conditions. Requests for data access will be evaluated on a case-by-case basis.
